# Correction: Seeking potent anti-tubercular agents: design and synthesis of substituted-*N*-(6-(4-(pyrazine-2-carbonyl)piperazine/homopiperazine-1-yl)pyridin-3-yl)benzamide derivatives as anti-tubercular agents

**DOI:** 10.1039/d0ra90059a

**Published:** 2020-05-19

**Authors:** Singireddi Srinivasarao, Adinarayana Nandikolla, Amaroju Suresh, Kevin Van Calster, Linda De Vooght, Davie Cappoen, Balaram Ghosh, Himanshu Aggarwal, Sankaranarayanan Murugesan, Kondapalli Venkata Gowri Chandra Sekhar

**Affiliations:** Department of Chemistry, Birla Institute of Technology and Science, Pilani Hyderabad Campus, Jawahar Nagar, Kapra Mandal Hyderabad-500078 Telangana India kvgc@hyderabad.bits-pilani.ac.in kvgcs.bits@gmail.com +91 40 66303527; Department of Green Chemistry and Technology, Faculty of Bioscience Engineering, Ghent University Coupure Links 653 Ghent B-9000 Belgium; Department of Pharmacy, Birla Institute of Technology and Science, Pilani, Hyderabad Campus Jawahar Nagar, Kapra Mandal Hyderabad-500078 Telangana India; Medicinal Chemistry Research Laboratory, Department of Pharmacy, Birla Institute of Technology and Science, Pilani 333031 India

## Abstract

Correction for ‘Seeking potent anti-tubercular agents: design and synthesis of substituted-*N*-(6-(4-(pyrazine-2-carbonyl)piperazine/homopiperazine-1-yl)pyridin-3-yl)benzamide derivatives as anti-tubercular agents’ by Singireddi Srinivasarao *et al.*, *RSC Adv.*, 2020, **10**, 12272–12288, DOI: 10.1039/D0RA01348J.

The authors regret that the name of one of the authors (Linda De Vooght) was shown incorrectly in the original article. The corrected author list is as shown above.

The Royal Society of Chemistry apologises for these errors and any consequent inconvenience to authors and readers.

## Supplementary Material

